# Taxonomic and phylogenetic contributions to Diatrypaceae from southeastern Tibet in China

**DOI:** 10.3389/fmicb.2023.1073548

**Published:** 2023-03-22

**Authors:** Hai-Xia Ma, Zhan-En Yang, Zi-Kun Song, Zhi Qu, Yu Li, An-Hong Zhu

**Affiliations:** ^1^Hainan Key Laboratory of Tropical Microbe Resources, Institute of Tropical Bioscience and Biotechnology, Hainan Institute for Tropical Agricultural Resources, Chinese Academy of Tropical Agricultural Sciences, Haikou, China; ^2^College of Biodiversity Conservation, Southwest Forestry University, Kunming, China; ^3^College of Plant Protection, Jilin Agricultural University, Changchun, China; ^4^Chinese Academy of Tropical Agricultural Sciences, Haikou, China

**Keywords:** Ascomycota, Diatrypaceous fungi, multigene phylogeny, taxonomy, wood-decaying fungi, Xylariales

## Abstract

In this study, we investigated the diversity of diatrypaceous fungi from southeastern Tibet in China. The phylogenetic analyses were carried out based on ITS and β-tubulin sequences of 75 taxa of Diatrypaceae from around the world. Based on a combination of morphological features and molecular evidence, a new genus—*Alloeutypa*, with three new species—*A. milinensis*, *Diatrype linzhiensis*, and *Eutypella motuoensis*, and a new combination—*A. flavovirens*, were revealed by the materials in China. *Alloeutypa* is characterized by stromatal interior olivaceous buff, stromata producing well-developed discrete, and ascospores allantoid, subhyaline. These characteristics separate the new genus from the similar genus *Eutypa*. Comprehensive morphological descriptions, illustrations, and a phylogenetic tree to show the placement of new taxa are provided. All novelties described herein are morphologically illustrated and phylogeny investigated to better integrate taxa into the higher taxonomic framework and infer their phylogenetic relationships as well as establish new genera and species. Our results indicate that the diatrypaceous fungi harbor higher species diversity in China.

## Introduction

Diatrypaceae Nitschke was introduced by Nitschke (1869) with *Diatrype* Fries as the type genus (Nitschke, 1869; Maharachchikumbura et al., 2015; Senanayake et al., 2015). Diatrypaceous taxa are abundant in Xylariales Nannf., which are widely distributed throughout the world, mostly saprophytic on dead or decaying angiosperms (Carter, 1991; Acero et al., 2004; Trouillas and Gubler, 2004; Trouillas et al., 2010a,b; Mehrabi et al., 2015; Konta et al., 2020; Yang et al., 2022), and some are pathogens or endophytes (Acero et al., 2004; de Errasti et al., 2014; Mehrabi et al., 2019; Konta et al., 2020; Dissanayake et al., 2021). In recent years, some new genera of the family Diatrypaceae have been reported combining morphological characteristics and multi-locus phylogeny (Dayarathne et al. 2016; Senwanna et al. 2017; Phookamsak et al. 2019; Dayarathne et al., 2020b). Hyde et al. (2020) compiled a taxonomic compilation of Sordariomycetes in which 20 genera of the family were listed; subsequently, the classification was followed by Wijayawardene et al. (2020). Dayarathne et al. (2020a) introduced a new genus, *Halocryptosphaeria* Dayarath., Devadatha, V.V. Sarma & K.D. Hyde saprophytic on decaying wood of *Avicennia marina* (Forsk.) Vierh. Konta et al. (2020) introduced a new genus, *Allodiatrype* Konta & K.D. Hyde, which included three new species and one new combination. Subsequently, *Paraeutypella* L.S. Dissan., J.C. Kang, Wijayaw. & K.D. Hyde, and *Pseudodiatrype* S.H. Long & Q.R. Li were introduced by Dissanayake et al. (2021) and Long et al. (2021), respectively, based on morphological distinctions and polygenic phylogenetic analyses.

The genus *Diatrype* Fr. was established by Fries (1849) and typified with *D. disciformis* (Hoffm.) Fr. The genus was characterized by stromata widely effuse or verrucose, flat or slightly convex, with discoid or sulcate ostioles at the surface, eight-spored and long-stalked asci and hyaline or brownish, allantoid ascospores (Rappaz, 1987; Vasilyeva and Stephenson, 2004; Vasilyeva and Stephenson, 2009; Senanayake et al., 2015). Recently, Zhu et al. (2021) included a new species, and Yang et al. (2022) introduced two new taxa with polysporous asci as members in *Diatrype* based on the phylogenies inferred from the dataset of ITS and β-tubulin.

*Eutypa* Tul. & C. Tul. was established by Tulasne and Tulasne (1863) based on *E. lata* (Pers.) Tul. & C. Tul. The genus is characterized by stromata which are irregular in shape, as confluent bumps, with conspicuous, scattered, roundish to prominent ostioles on the host surface, 8-spore asci with indistinct apical rings, and ascospores allantoid to ellipsoidal, aseptate, and pale yellowish (Hyde et al., 2020). Some species of this genus are disease-causing pathogens, for example, *E. lata* has been reported to cause dieback and canker in *Vitis vinifera* (grapevine; Moller and Kasimatis, 1978), *Prunus armeniaca* (apricots; Carter, 1957), and *Prunus salicina* (Carter, 1982); *E. leptoplaca* has been reported to be pathogenic to grapevine (Trouillas and Gubler, 2004).

The genus *Eutypella* (Nitschke) Sacc., established by Saccardo (1875) with *El. cerviculata* (Fr.) Sacc. as the type (Saccardo, 1882; Mehrabi et al., 2019; Hyde et al., 2020), which includes 111 morphological species (Species Fungorum 2020), and only 17 species have sequence data (Hyde et al., 2020). *Eutypella* taxa have a wide host range, and some species are phytopathogens that cause canker, such as *El. parasitica* R.W. Davidson & R.C. Lorenz causes canker in *Acer* spp. (Kowalski and Bednarz, 2017), *El. microtheca* Trouillas, W.M. Pitt & Gubler causes canker in *Vitis vinifera,* and *Prunus* spp. (Trouillas et al., 2011; Moyo et al., 2018a,b). The important characteristics of this genus are valsoid configuration stromata, usually comprising host tissues or a mixture of host and fungal tissues, mostly sulcate, sometimes rounded ostioles, converging ostiolar necks, eight-spored asci, and allantoid ascospores (Glawe and Rogers, 1984; Vasilyeva and Stephenson, 2006; Hyde et al., 2020). Rappaz (1987) made a taxonomic revision of Diatrypaceae, in which 76 taxa of *Eutypella* were described. Afterward, Carmarán et al. (2006) performed a phylogenetic analysis of Diatrypaceae based on ascus morphology and other morphological characteristics and transferred six species from *Eutypella* to *Peroneutypa* Berl. Dissanayake et al. (2021) transferred *El. citricola* Speg. and *El. vitis* (Schwein.) Ellis & Everh. to *Paraeutypella* combining morphological and phylogenetic data.

*Diatrype*, *Eutypa,* and *Eutypella* are all unresolved lineages, and phylogenetic studies indicated that the three genera do not form monophyletic groups, even though they clustered within Diatrypaceae (Hyde et al., 2020; Wijayawardene et al., 2020; Long et al., 2021; Yang et al., 2022). In an investigation of the diversity of wood-decaying fungi in southeastern Tibet of China, three undescribed species of diatrypaceous fungi were collected. In order to further the knowledge of species diversity and taxonomy of Diatrypaceae, we carried out complete morphological and molecular phylogenetic studies on these specimens with an emphasis on diatrypaceous fungi. In this study, we introduce a new genus, three new species, and a new combination of Diatrypaceae occurring on decaying wood.

## Materials and methods

### Specimen collection

The specimens studied in this article were collected from Motuo County and Milin County in Linzhi City of southeastern Tibet, China. *In situ* photographs of the specimens were taken with a Canon G16 camera (Tokyo, Japan). Fresh specimens were dried and deposited following Yang et al. (2022).

### Morphological examination

The studied specimens were macromorphologically observed with the aid of a VHX-600E microscope of Keyence Corporation (Osaka, Japan) up to ×200. The microscopic procedure followed Song et al. (2022). Specimen sections were mounted in water, 10% potassium hydroxide (KOH), and Melzer’s reagent (1.5 g potassium iodide, 0.5 g crystalline iodine, and 22 g chloral hydrate dissolved in 20 ml distilled water), and then microscopic examinations were carried out with an Olympus IX73 inverted fluorescence microscope (Tokyo, Japan) at magnifications up to × 1,000.

### DNA Extraction, PCR Amplification, and Sequencing

Genomic DNA was extracted from dried specimens using CTAB rapid plant genome extraction kit-DN14 (Aidlab Biotechnologies Co., Ltd., Beijing, China) and RaPure Plant DNA Mini Kit (Magen Biotechnology) according to the manufacturer’s instructions. The internal transcribed spacer (ITS) region and β-tubulin (TUB2) were amplified with primer pairs ITS5/ITS4 (White et al., 1990) and T1/T22 (O'Donnell and Cigelnik, 1997), respectively. Polymerase chain reaction (PCR) was performed following Song et al. (2022). DNA sequencing was performed at BGI tech, Guangzhou, China. All newly generated sequences in this study including eight ITS sequences and six β-tubulin sequences were deposited in GenBank (Table 1).^1^

**Table 1:** List of species, specimens, and GenBank accession numbers of sequences used in this study.

Species	Strain	Host/substrate	Origin	GenBank accession numbers		References
				ITS	TUB2	
*Allocryptovalsa cryptovalsoidea*	HVFIG 05	*Ficus carica*	Australia	HQ692574	HQ692525	Trouillas et al. (2011)
*Allocryptovalsa elaeidis*	MFLUCC 15–0707	*Elaeis guineensis*	Thailand	MN308410	MN340296	Konta et al. (2020)
*Allocryptovalsa rabenhorstii*	WA07CO	*Vitis vinifera*	Australia	HQ692620	HQ692522	Trouillas et al. (2011)
*Allocryptovalsa rabenhorstii*	WA08CB	*Vitis vinifera*	Australia	HQ692619	HQ692523	Trouillas et al. (2011)
*Allodiatrype arengae* ^T^	MFLUCC 15–0713	*Arenga pinnata*	Thailand	MN308411	MN340297	Konta et al. (2020)
*Allodiatrype elaeidicola*	MFLUCC 15-0737a	*Elaeis guineensis*	Thailand	MN308415	MN340299	Konta et al. (2020)
*Allodiatrype elaeidis*	MFLUCC 15-0708a	*Elaeis guineensis*	Thailand	MN308412	MN340298	Konta et al. (2020)
*Alloeutypa flavovirens*	**E48C, CBS 272.87**	***Quercus ilex***	**France**	**AJ302457**	**DQ006959**	Rolshausen et al. (2006)
*Alloeutypa flavovirens*	**MFLU 19–0911**	***Quercus* sp. *(Fagaceae)***	**Italy**	**MZ456005**	**MZ476771**	Boonmee et al. (2021)
***Alloeutypa milinensis***^T^	**FCATAS 4309**	**unidentified dead wood**	**China**	**OP538689**	**OP557595**	**This study**
***Alloeutypa milinensis***^T^	**FCATAS 4382**	**unidentified dead wood**	**China**	**OP538690**	**OP557596**	**This study**
*Anthostoma decipiens* ^T^	JL567	*Vitis vinifera*	Spain	JN975370	JN975407	Luque et al. (2012)
*Anthostoma decipiens* ^T^	CD	*Carpinus betulus*	Austria	KC774565	NA	Jaklitsch et al. (2014)
*Cryptosphaeria eunomia* var. *fraxini*	C1C (CBS 216.87)	*Fraxinus excelsior*	Switzerland	AJ302417	NA	Acero et al. (2004)
*Cryptosphaeria eunomia* var. *fraxini*	CBS223.87	*Fraxinus excelsior*	Switzerland	AJ302421	NA	Acero et al. (2004)
*Cryptosphaeria ligniota*	CBS 273.87	*Populus tremula*	Switzerland	KT425233	KT425168	Acero et al. (2004)
*Cryptosphaeria pullmanensis*	ATCC 52655	NA	Washington, USA	KT425235	KT425170	Trouillas et al. (2015)
*Cryptosphaeria subcutanea*	CBS 240.87	NA	Norway	KT425232	KT425167	Trouillas et al. (2015)
*Cryptovalsa ampelina*	A001	NA	Australia	GQ293901	GQ293972	Trouillas et al. (2010b)
*Cryptovalsa ampelina*	DRO101	NA	USA	GQ293902	GQ293982	Trouillas et al. (2010b)
*Diatrype betulaceicola*	FCATAS 2725	*Betula* sp.	China	OM040386	OM240966	Yang et al. (2022)
*Diatrype betulaceicola*	FCATAS 2726	*Betula* sp.	China	OM040387	OM240967	Yang et al. (2022)
*Diatrype betulae*	CFCC 52416	*Betula davurica*	China	MW632943	NA	Zhu et al. (2021)
*Diatrype bullata*	UCDDCh400	NA	United States	DQ006946	DQ007002	Rolshausen et al. (2006)
*Diatrype bullata*	D6C	*Salix* sp.	Switzerland	AJ302422	NA	Acero et al. (2004)
*Diatrype castaneicola*	CFCC 52425	*Castanea mollissima*	China	MW632941	NA	Zhu et al. (2021)
*Diatrype castaneicola*	CFCC 52426	*Castanea mollissima*	China	MW632942	NA	Zhu et al. (2021)
*Diatrype disciformis* ^T^	CBS 205.87	*Fagus sylvatica*	Switzerland	AJ302437	NA	Acero et al. (2004)
*Diatrype disciformis* ^T^	GNA14	*Fagus grandifolia*	United States	KR605644.1	KY352434.1	Senanayake et al. (2015)
*Diatrype enteroxantha*	HUEFS155114	NA	Brazil	KM396617	KT003700	de Almeida et al. (2016)
*Diatrype enteroxantha*	HUEFS155116	NA	Brazil	KM396618	KT022236	de Almeida et al. (2016)
*Diatrype iranensis* (*Diatrypella iranensis*)	IRAN 2280C	*Quercus brantii*	Iran	KM245033	KY352429	Mehrabi et al. (2015)
*Diatrype lancangensis*	GMB0045	unidentified dead wood	China	MW797113	MW814885	Long et al. (2021)
*Diatrype lancangensis*	GMB0046	unidentified dead wood	China	MW797114	MW814886	Long et al. (2021)
*Diatrype larissae*	FCATAS 2723	dead wood	China	OM040384	OM240964	Yang et al. (2022)
*Diatrype larissae*	FCATAS 2724	dead wood	China	OM040385	OM240965	Yang et al. (2022)
*Diatrype lijiangensis*	MFLU 19–0717	dead wood	China	MK852582	MK852583	Thiyagaraja et al. (2019)
***Diatrype linzhiensis***	**FCATAS 4304**	**unidentified dead wood**	**China**	**OP538691**	**OP557597**	**This study**
***Diatrype linzhiensis***	**FCATAS 4381**	**unidentified dead wood**	**China**	**OP538692**	**OP557598**	**This study**
*Diatrype macrospora* (*Diatrypella macrospora*)	IRAN 2344C	*Quercus brantii*	Iran	KR605648	KY352430	Mehrabi et al. (2015)
*Diatrype palmicola*	MFLUCC 11-0018	*Caryota urens*	Thailand	KP744438	NA	Liu et al. (2015)
*Diatrype palmicola*	MFLUCC 11-0020	*Caryota urens*	Thailand	KP744439	NA	Liu et al. (2015)
*Diatrype quercicola*	CFCC 52418	*Quercus mongolica*	China	MW632938	MW656386	Zhu et al. (2021)
*Diatrype quercicola*	CFCC 52419	*Quercus mongolica*	China	MW632939	MW656387	Zhu et al. (2021)
*Diatrype quercina* (*Diatrypella quercina*)	F-091966	*Quercus faginea*	Spain	AJ302444	NA	Acero et al. (2004)
*Diatrype spilomea*	CBS 212.87	*Acer campestre*	Switzerland	AJ302433	NA	Acero et al. (2004)
*Diatrype stigma*	DCASH200	*Quercus* sp.	USA	GQ293947	GQ294003	Trouillas et al. (2010b)
*Diatrype stigma*	UCD23-Oe	*Olea europaea*	NA	JX515704	JX515670	Úrbez-Torres et al. (2013)
*Diatrype undulata*	CBS 271.87	*Betula* sp.	Switzerland	AJ302436	NA	Acero et al. (2004)
*Diatrype undulata*	Olrim324	*Betula pendula*	Lithuania	AY354239	NA	Lygis et al. (2004)
*Diatrype virescens*	CBS 128344	NA	USA	MH864890	NA	Vu et al. (2019)
*Diatrype whitmanensis*	CDB011	*Vitis vinifera*	USA	GQ293954	GQ294010	Trouillas et al. (2010b)
*Diatrype whitmanensis*	DCHES100	*Aesculus californica*	USA	GQ293951	GQ294008	Trouillas et al. (2010b)
*Diatrypella atlantica*	HUEFS 136873	unidentified plant	Brazil	KM396614	KR259647	de Almeida et al. (2016)
*Diatrypella atlantica*	HUEFS 194228	unidentified plant	Brazil	KM396615	KR363998	de Almeida et al. (2016)
*Diatrypella delonicis*	MFLU 16-1032	*Delonix regia*	Thailand	MH812995	MH847791	Hyde et al. (2020)
*Diatrypella delonicis*	MFLUCC 15-1014	*Delonix regia*	Thailand	MH812994	MH847790	Hyde et al. (2019)
*Diatrypella favacea*	Islotate 380	NA	USA	KU320616	NA	de Almeida et al. (2016)
*Diatrypella heveae*	MFLUCC 17-0368	*Hevea brasiliensis*	Thailand	MF959501	MG334557	Senwanna et al. (2017)
*Diatrypella pulvinate*	H048	*Salix alba*	Czech Republic	FR715523	FR715495	de Almeida et al. (2016)
*Diatrypella verruciformis* ^T^	UCROK1467	*Quercus agrifolia*	USA	JX144793	JX174093	Lynch et al. (2013)
*Diatrypella verruciformis* ^T^	UCROK754	*Quercus agrifolia*	USA	JX144783	JX174083	Lynch et al. (2013)
*Diatrypella vulgaris*	HVFRA02	*Fraxinus angustifolia*	Australia	HQ692591	HQ692503	Trouillas et al. (2011)
*Diatrypella vulgaris*	HVGRF03	*Citrus paradisi*	Australia	HQ692590	HQ692502	Trouillas et al. (2011)
*Eutypa astroidea*	E49C, CBS 292.87	*Fraxinus excelsior*	Switzerland	AJ302458	DQ006966	Rolshausen et al. (2006)
*Eutypa cerasi*	GMB0048	unidentified plant	China	MW797104	MW814893	Long et al. (2021)
*Eutypa cremea*	STEU 8082	*Vitis vinifera*	South Africa	KY111656	KY111598	Moyo et al. (2018b)
*Eutypa cremea*	STEU 8410	*Prunus armeniaca*	South Africa	KY752765	KY752789	Moyo et al. (2018b)
*Eutypa crustata*	CBS 210.87	*Ulmus* sp.	France	AJ302448	DQ006968	Rolshausen et al. (2006)
*Eutypa laevata*	CBS 291.87	*Salix* sp.	Switzerland	HM164737	HM164771	Trouillas and Gubler (2010)
*Eutypa lata* ^T^	EP18	*Vitis vinifera*	New South Wales	HQ692611	HQ692501	Trouillas et al. (2011)
*Eutypa lata* (*Eutypa armeniacae*) ^T^	CBS 622.84	*Vitis vinifera*	Italy	AJ302446	DQ006964	Acero et al. (2004), Rolshausen et al. (2006)
*Eutypa lata* ^T^	ATCC 28120	*Prunus armeniaca*	Australia	DQ006948	DQ006975	Rolshausen et al. (2006)
*Eutypa lejoplaca*	CBS 248.87	*Acer pseudoplatanus*	Switzerland	DQ006922	DQ006974	Rolshausen et al. (2006)
*Eutypa leptoplaca*	CBS 287.87	*Frangula alnus*	Switzerland	DQ006924	DQ006961	Rolshausen et al. (2006)
*Eutypa maura*	CBS 219.87	*Acer pseudoplatanus*	Switzerland	DQ006926	DQ006967	Rolshausen et al. (2006)
*Eutypa petrakii* var. *hederae*	CBS 285.87	NA	Switzerland	MH862077	NA	Vu et al. (2019)
*Eutypa petrakii* var. *petrakii*	CBS 244.87	*Prunus spinosa*	Switzerland	AJ302455	DQ006958	Acero et al. (2004), Rolshausen et al. (2006)
*Eutypella cearensis*	HUEFS 131070	*unidentified plant*	Brazil	KM396639	NA	de Almeida et al. (2016)
*Eutypella cerviculata*	EL59C	*Alnus glutinosa*	Switzerland	AJ302468	NA	Acero et al. (2004)
*Eutypella cerviculata*	M68	*Alnus glutinosa*	Latvia	JF340269	NA	Arhipova et al. (2012)
*Eutypella leprosa*	EL54C	*Tilia* sp.	Switzerland	AJ302463	NA	Acero et al. (2004)
*Eutypella leprosa*	Isolate 60	NA	USA	KU320622	NA	de Almeida et al. (2016)
***Eutypella motuoensis***	**FCATAS 4035**	**unidentified dead wood**	**China**	**OP538695**	**NA**	**This study**
***Eutypella motuoensis***	**FCATAS 4082**	**unidentified dead wood**	**China**	**OP538693**	**OP557599**	**This study**
***Eutypella motuoensis***	**FCATAS 4378**	**unidentified dead wood**	**China**	**OP538696**	**NA**	**This study**
***Eutypella motuoensis***	**FCATAS 4379**	**unidentified dead wood**	**China**	**OP538694**	**OP557600**	**This study**
*Eutypella microtheca*	ADEL200	*Ulmus procera*	Australia	HQ692559	HQ692527	Trouillas et al. (2011)
*Eutypella microtheca*	BCMX01	Cabernet-Sauvignon grapevine	Mexico	KC405563	KC405560	Paolinelli-Alfonso et al. (2015)
*Eutypella parasitica*	CBS 210.39	NA	USA	MH855984	NA	Vu et al. (2019)
*Eutypella parasitica*	TO1/1	*Acer pseudoplatanus*	Slovenia	AM295770	NA	Piškur et al. (2007)
*Eutypella persica*	IRAN 2540C	*Alnus* sp.	Iran	KX828144	KY352451	Mehrabi et al. (2019)
*Eutypella quercina*	IRAN 2543C	*Quercus* sp.	Iran	KX828139	KY352449	Mehrabi et al. (2019)
*Eutypella semicircularis*	MP4669	*Alnus acuminata*	Panama	JQ517314	NA	Chacón et al. (2013)
*Halodiatrype avicenniae*	MFLUCC 15-0953	*Avicennia* sp.	Thailand	KX573916	KX573931	Dayarathne et al. (2016)
*Halodiatrype salinicola* ^T^	MFLUCC 15-1,277	submerged marine wood	Thailand	KX573915	KX573932	Dayarathne et al. (2016)
*Kretzschmaria deusta*	CBS 826.72	*Fagus sylvatica*	Belgium: Mechelen	KU683767	KU684190	U’ren et al. 2016
*Monosporascus cannonballus* ^T^	ATCC 26931	NA	USA	FJ430598	NA	Unpublished
*Monosporascus cannonballus* ^T^	CMM 3646	*Boerhavia* sp.	Brazil	JX971617	NA	Sales et al. (2010)
*Neoeutypella baoshanensis* ^T^	GMB0052	unidentified plant	China	MW797106	MW814878	Long et al. (2021)
*Neoeutypella baoshanensis* ^T^	HMAS 255436	*Pinus armandii*	China	MH822887	MH822888	Phookamsak et al. (2019)
*Paraeutypella citricola*	HVVIT07	*Vitis vinifera*	Australia	HQ692579	HQ692512	Trouillas et al. (2011)
*Paraeutypella citricola*	HVGRF01	*Citrus paradisi*	Australia	HQ692589	HQ692521	Trouillas et al. (2011)
*Paraeutypella vitis*	UCD2291AR	*Vitis vinifera*	USA	HQ288224	HQ288303	Úrbez-Torres et al. (2012)
*Paraeutypella vitis*	UCD2428TX	*Vitis vinifera*	Texas, USA	FJ790851	GU294726	Úrbez-Torres et al. (2012)
*Peroneutypa curvispora*	HUEFS 136877	NA	Brazil	KM396641	NA	de Almeida et al. (2016)
*Peroneutypa rubiformis*	MFLUCC 17-2,142	NA	Thailand	MG873477	NA	Shang et al. (2018)
*Peroneutypa scoparia*	MFLUCC 11-0478	*bamboo*	Thailand	KU940151	NA	Dai et al. (2016)
*Pseudodiatrype hainanensis* ^T^	GMB0054	unidentified plant	China	MW797111	MW814883	Long et al. (2021)
*Pseudodiatrype hainanensis* ^T^	GMB0055	unidentified plant	China	MW797112	MW814884	Long et al. (2021)
*Pedumispora rhizophorae* ^T^	BCC44877	*Rhizophora apiculata*	Thailand	KJ888853	NA	Klaysuban et al. (2014)
*Pedumispora rhizophorae* ^T^	BCC44878	*Rhizophora apiculata*	Thailand	KJ888854	NA	Klaysuban et al. (2014)
*Quaternaria quaternate*	GNF13	*Fagus* sp.	Iran	KR605645	KY352464	Mehrabi et al. (2015)
*Quaternaria quaternate*	CBS 278.87	*Fagus sulvatica*	Switzerland	AJ302469	NA	Acero et al. (2004)
*Xylaria hypoxylon*	CBS 122620	NA	Sweden	AM993141	KX271279	Peršoh et al. (2009)

NA: not applicable; T: type species of the genus. Newly generated sequences are indicated in bold.

### Phylogenetic analyses

Sequencher 4.6 (GeneCodes, Ann Arbor, MI, United States) was used to edit the DNA sequence. Sequences were manually cut and orientation adjusted using BioEdit software (Hall, 1999). Sequences were aligned using the “G-INS-i” strategy at the MAFFT 7 (http://mafft.cbrc.jp/alignment/server/) website and manually corrected using BioEdit. The sequences of *Kretzschmaria deusta* (Hoffm.) P.M.D. and *Xylaria hypoxylon* (L.) Grev. were obtained from GenBank as out-groups.

Maximum likelihood analyses were performed in raxmlGUI 2.0 selecting ML + rapid bootstrap analysis and GTRGAMMA+G as the surrogate model (Ma et al., 2022; Song et al., 2022). Branch support (BS) for ML analysis was determined by 1,000 bootstrap replicates. MrModeltest 2.3 (Nylander, 2004) was used to determine the best-fit evolution model for each dataset for Bayesian inference (BI). Bayesian inference was calculated with MrBayes 3.1.2 with a general time reversible (GTR + I + G) model of DNA substitution and a gamma distribution rate variation across sites (Ronquist and Huelsenbeck, 2003). Four simultaneous Markov chains were run for 2000000 generations, and every 100 generations were sampled as a tree. The first one-fourth generations were discarded as burn-in. The majority rule consensus tree of all remaining trees is computed. Branches were considered as significantly supported if they received maximum likelihood bootstrap (BS) ≥ 70% and Bayesian posterior probabilities (BPP) ≥ 0.95.

## Results

### Molecular phylogeny

**Figure 1:**
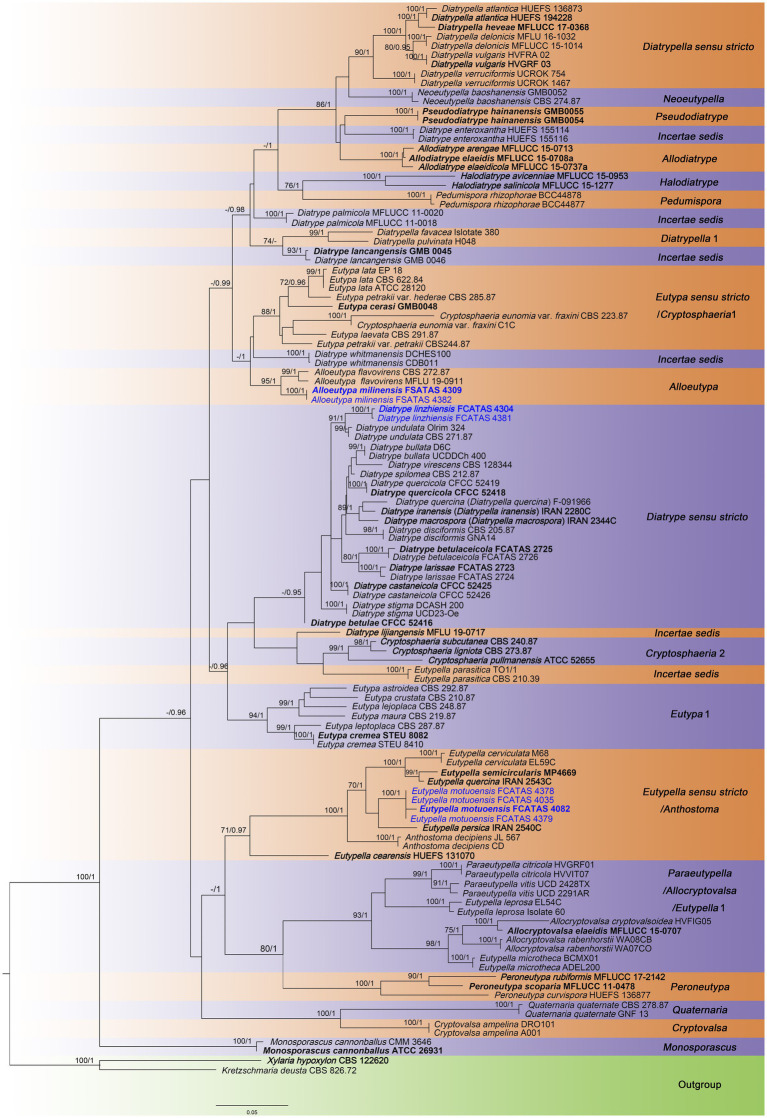
Phylogram generated from maximum likelihood (RA × ML) analyses, based on ITS-β-tubulin matrix. Branches are labeled with maximum likelihood bootstrap ≥ 70% and Bayesian posterior probabilities ≥ 0.95. Ex-type strains are in bold. Newly generated strains are in blue. Bold branches indicate that the length has been cut in half.

The contribution of the molecular phylogenetic tree based on 197 sequences of two DNA loci (116 ITS and 81 β-tubulin sequences) was composed of 116 samples representing 75 strains of Diatrypaceae (Table 1). The concatenated dataset had an aligned length of 1936 characteristics, including gaps (609 for ITS and 1,327 for TUB2). Bayesian obtained a topology similar to ML, with an average standard deviation of split frequencies = 0.007766 (BI). Only the ML tree is provided in Figure 1 with the likelihood bootstrap values (≥ 70%, before the slash) and Bayesian posterior probabilities (≥ 0.95, behind the slash) labeled along the branches.

The topology of the phylogenetic tree is similar to those in previous studies (Konta et al., 2020; Zhu et al., 2021). For the in-groups, species from 18 genera were distributed in 24 clades: including 18 main clades, *Diatrypella sensu stricto*, *Neoeutypella*, *Pseudodiatrype*, *Allodiatrype*, *Halodiatrype*, *Pedumispora*, *Diatrypella* 1, *Eutypa sensu stricto*/*Cryptosphaeria* 1, *Alloeutypa*, *Diatrype sensu stricto*, *Cryptosphaeria* 2, *Eutypa* 1, *Eutypella sensu stricto*/*Anthostoma*, *Paraeutypella*/*Allocryptovalsa*/*Eutypella* 1, *Peroneutypa*, *Quaternaria*, *Cryptovalsa*, *Monosporascus*, and six incertae sedis clades (*Diatrype enteroxantha*, *D. lancangensis*, *D. lijiangensis*, *D. palmicola*, *D. whitmanensis*, and *Eutypella parasitica*). *Allodiatrype*, *Alloeutypa*, *Monosporascus*, *Neoeutypella*, *Paraeutypella*, *Peroneutypa*, and *Pseudodiatrype* were shown to be monophyletic and well-supported in our tree. *Halodiatrype* and *Pedumipora*, *Cryptovalsa* and *Quaternaria* formed a strongly supported claded respectively. *Anthostoma decipiens* (JL567 and CD) grouped together is sister to *Eutypella sensu stricto* with strong support (ML/BI = 100/1). *Eutypella leprosa*, *El*. *microtheca*, and several species from *Paraeutypella* and *Allocryptovalsa* formed a large clade with relatively strong support. The new genus *Alloeutypa* included two species, *A. milinensis* and *A. flavovirens*, formed a distinct clade. The other two new species—*Diatrype linzhiensis* and *Eutypella motuoensis*, formed distinct lineages in the tree. Some confused taxa, for example, *Diatrype enteroxantha*, *D. lancangensis, D. lijiangensis*, *D. palmicola*, *D. whitmanensis*, and *Eutypella parasitica*, formed a single clade or mixed with other genera.

### Taxonomy

*Alloeutypa* Hai X. Ma, Z.E. Yang & Y. Li, gen. Nov.

MycoBank: 846109.

Etymology: referring to the morphological resemblance to *Eutypa*.

*Descriptions—*Saprobic on dead angiosperm branch. Sexual morph: Stromata scattered on the host, pustulate, solitary or aggregated, superficial, irregularly shaped or oblong to strip, upper surface flat to slightly curved; surface black, with numerous ascomata in a single stroma. Endostroma consists of outer layer of black, small, dense, thin parenchymal cells and inner layer of olivaceous buff, large, loose parenchymal cells. Ostioles opening to outer surface, appearing as black spots, separately, papillate or apapillate. Perithecium globose to subglobose, individual ostiole with a neck. Peridium composed of outer layer of dark brown to brown, thin-walled cells, inner layer of hyaline thin-walled cells. Paraphyses elongate, hyaline, long, filiform, unbranched, septate, guttulate. Asci eight-spored, unitunicate, clavate, long-stalked, apically rounded. Ascospores irregularly arranged, allantoid, aseptate, slightly curved, subhyaline to yellowish, smooth-walled, several oil droplets in each end.

Type species: *Alloeutypa milinensis* Hai X. Ma, Z.E. Yang & Y. Li.

Notes: In the phylogenetic tree (Figure 1), *Eutypa* species are distributed in two distinct clades *Eutypa sensu stricto* and *Eutypa* 1, indicating that the genus is polyphyletic. The type species, *E. lata* clusters in *Eutypa* clade1 which can be regarded as *Eutypa sensu stricto*. However, it is hard to justify *Eutypa* 1 as a new genus without examining old types of species and identified fresh collections with molecular data.

The sexual morphology of *Eutypa sensu stricto* (as *Eutypa* taxonomic species 2) comprises wide-spreading stromata that embedded in decorticated wood or bark, usually poorly developed with ill-defined margins, surface black, interior white or blackened, eight-spore asci spindle-shaped, long-stipitate, ascospores allantoid, subolivaceous (Glawe and Rogers, 1984). The Chinese collection in this study is clearly different from members of *Eutypa sensu stricto* based on the green interior of the stromata, discrete, Diatrype-like.

Based on the morpho-molecular differences, the new genus *Alloeutypa* is introduced to accommodate *Alloeutypa milinensis*. *Alloeutypa* is typified by *A. milinensis*, which was found on dead branches of angiosperm plant from southeastern Tibet in China. *Eutypa flavovirens* resembles *A. milinensis* in having well-developed discrete, Diatrype-like stromata with yellow-green to olive-green interior tissue, asci spindle-shaped, long-stipitate, ascospores allantoid, and subhyaline to subolivaceous. The phylogenetic analyses based on ITS and β-tubulin sequence data also supported *Alloeutypa* as a monophyletic genus in the Diatrypaceae, and *A. milinensis* and *A. flavovivens* as separate lineages within *Alloeutypa*. Thus, based on morphological evidence and phylogenetic analyses, we accommodate *Alloeutypa* as a new genus with *A. milinensis* as the type, and *E. flavovirens* was transferred to *Alloeutypa* as *A. flavovirens* comb. nov.

**Figure 2:**
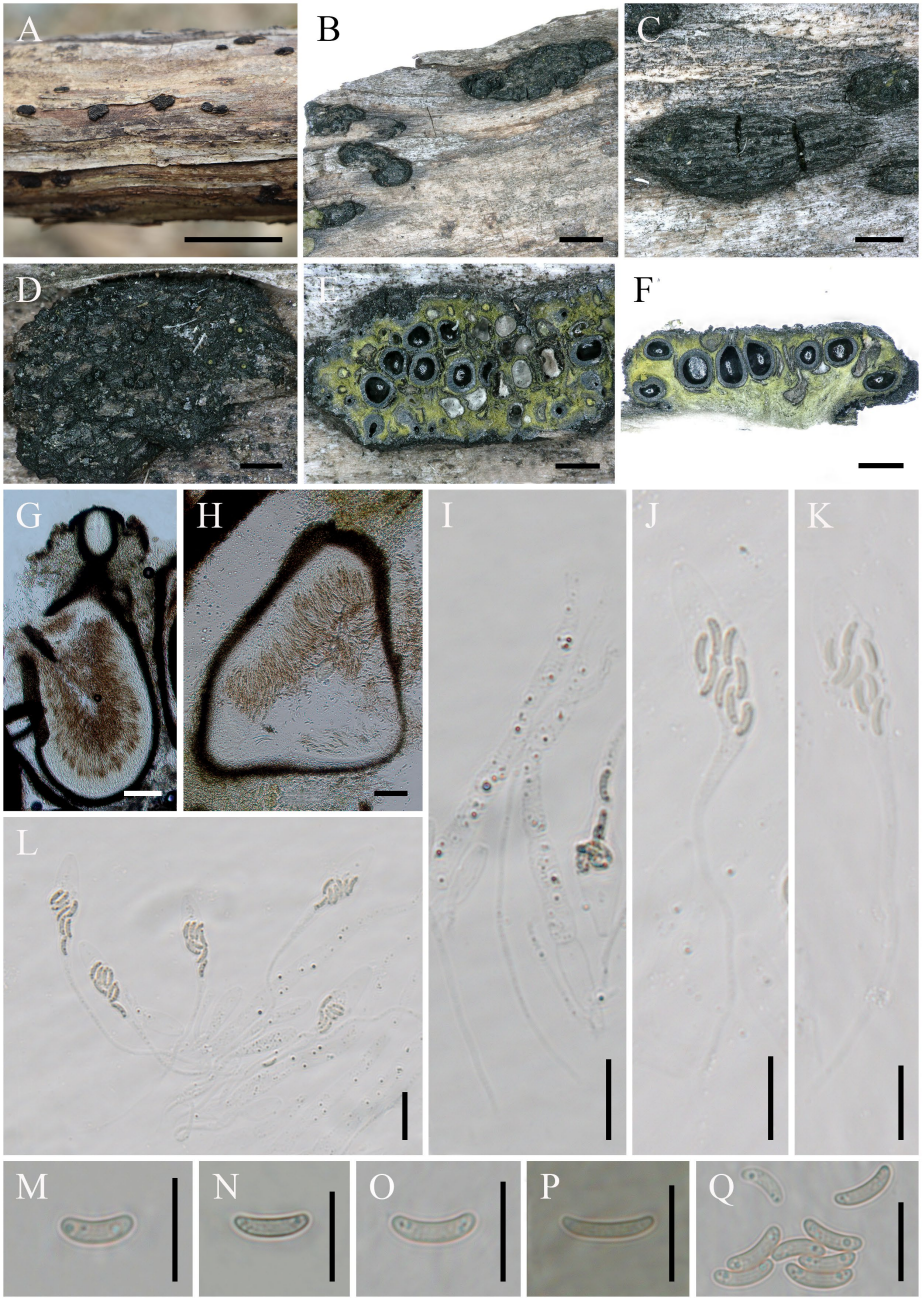
*Alloeutypa milinensis* (FCATAS 4309, Holotype). **(A–D)** Stromata on substrate. **(E)** Cross section of a stroma. **(F,G)** Vertical section through stroma showing ostiole and perithecia. **(H)** Peridium. **(I)** Paraphyses. **(J–L)** Asci. **(M–Q)** Ascospores. Scale bars: **(A)** = 15 mm; **(B)** = 2 mm; **(C)** = 1 mm; **(D–F)** = 500 μm; **(G,H)** = 100 μm; **(I–L)** = 20 μm; **(M–Q)** = 10 μm.

*Alloeutypa milinensis* Hai X. Ma, Z.E. Yang & Y. Li, sp. *nov*. (Figure 2).

MycoBank: MB 846111.

*Type*: China. Tibet Autonomous Region, Linzhi City, Milin County, Pai Town, 29°30′2′ N, 94°50′26′ E, alt. 998 m, saprobic on dead branch, 7 October 2021, Haixia Ma, FCATAS 4309 (holotype).

*Etymology*: referring to the locality (Milin County) of the type specimens.

*Descriptions*: Saprobic on dead branches of unidentified plant. Sexual morph: Stromata scattered on the host, pustulate, solitary, superficial, 2–7.3 mm long × 0.9–2.2 mm broad (x̄ = 3.6 × 1.5 mm, *n* = 20), oblong to strip, upper surface flat to slightly curved; surface black with 14–50 perithecia immersed in stroma. Endostroma consists of outer layer of black, small, dense, thin parenchymal cells and inner layer of olivaceous buff, large, loose parenchymal cells, near base, whitish yellow-green. Ostioles opening to outer surface, appearing as black spots, separately, papillate or apapillate. Perithecium globose to subglobose, 261.2–512.2 μm high × 245.7–443.3 μm diam (x̄ = 383.8 × 334.1 μm, *n* = 30), individual ostiole with a neck. Peridium composed of outer layer of dark brown to brown, thin-walled cells, inner layer of hyaline thin-walled cells. Paraphyses elongate, hyaline, long, filiform, unbranched, septate, guttulate. Asci 97–194 × 7.5–16.7 μm (x̄ = 132.8 × 11.3 μm, *n* = 50), eight-spored, unitunicate, clavate, long-stalked (30–131.5 μm), apically rounded. Ascospores 6.6–10.1 × 1.7–2.6 μm (x̄ = 8.5 × 2.1 μm, *n* = 50), overlapping, allantoid, aseptate, slightly curved, subhyaline, smooth-walled, usually with two oil droplets.

*Asexual morph*: Undetermined.

*Additional specimen examined.*—China. Tibet Autonomous Region, Linzhi City, Milin County, Pai Town, 29°29′57′ N, 94°50′29′ E, alt. 996 m, saprobic on dead branch, 7 October 2021, Haixia Ma, FCATAS 4382.

Note: *Alloeutypa milinensis* grouped with *A. flavovirens* (*E. flavovirens*) based on the combined ITS + β-tubulin sequence data. In recent years, *A. flavovirens* (*E. flavovirens*) has been successively recorded in Thailand, India, and Italy, and the specimens from the three regions have some differences in morphology. Morphologically, the specimens of *A. flavovirens* (*E. flavovirens*) in Thailand differ from *A. milinensis* in smaller stromata (1–1.5 mm wide) and smaller perithecium diam (120–210 μm diam; Senanayake et al., 2015); the specimens from India differ by the smaller perithecium (212.5–396 × 184.6–363 μm), fewer perithecium in a stroma (2–12), and shorter ascus (75–110 × 6.1–8.8 μm; Niranjan et al., 2018); the specimen from Italy differs in having gregarious, aggregates to discrete stromata, smaller in size (0.7–1 mm diam), and smaller ascus (80–120 × 8–10 μm; Boonmee et al., 2021).

The sequence comparison showed that there are 97.22 and 95%, respectively, similarities in ITS and TUB2 between *A. milinensis* from China (FCATAS 4309) and *A. flavovirens* (*E. flavovirens*) from Italy (MFLU19-0911), while 97.13 and 94.12 between *A. milinensis* from China (FCATAS 4309) and *A. flavovirens* (*E. flavovirens*) from France (E48C, CBS 272.87). Unfortunately, TUB2 sequences of the Indian and Thailand collections are not available in GenBank. However, the ITS sequence comparison showed that there are both 92% similarities between *A. milinensis* from China (FCATAS 4309) and *A. flavovirens* (*E. flavovirens*) from India (PUFNI 310) and Thailand (MFLUCC 13-0625). Therefore, we described the Chinese material as a new species.

*Alloeutypa flavovirens*: (Pers.) Hai X. Ma & Z.E. Yang, comb. nov.

MycoBank: 846128.

Synonyms: *Sphaeria flavovirens* Pers., Syn. meth. Fung. (Göttingen) 1: 22, 1801. *Diatrype flavovirens* (Pers.) Fr., Summa veg. Scand., Sectio Post. (Stockholm): 385, 1849. *Eutypa flavovirens* (Pers.) Tul. & C. Tul., Select. fung. Carpol. (Paris) 2: 57, 1863.

Notes: *Alloeutypa flavovirens* is one of the most common fungi and found throughout the world and appears to have a wide host range (Glawe and Rogers, 1982, 1984; Rappaz, 1987). It is characterized by having yellow-greenish stromatic tissues, spindle-shaped asci with refractive apical invaginations, allantoid ascospores subhyaline to subolivaceous (Glawe and Rogers, 1984). It is most similar to *A. milinensis* in having the green interior of the stromata. There are no sequence data for the type of *A. flavovirens*, but there are two putatively named collections, CBS 272.87 and MFLU 19-0911, from France and Italy, respectively (Rolshausen et al. 2006; Boonmee et al., 2021). Based on the morphological and molecular analyses that the two collections were the records of *A. flavovirens* (*E. flavovirens*) by Senanayake et al. (2015) and Boonmee et al. (2021), in our phylogenetic tree, the two strains of *A. flavovirens* (*E. flavovirens*) clustered together with *A. milinensis* with strong support (95% ML, 1.00 BYPP; Figure 1) and maybe the same genus because of its location. However, morphological differences on size of stromata, perithecium, and ascus can distinguish the two species from each other (Senanayake et al., 2015; Boonmee et al., 2021).

**Figure 3:**
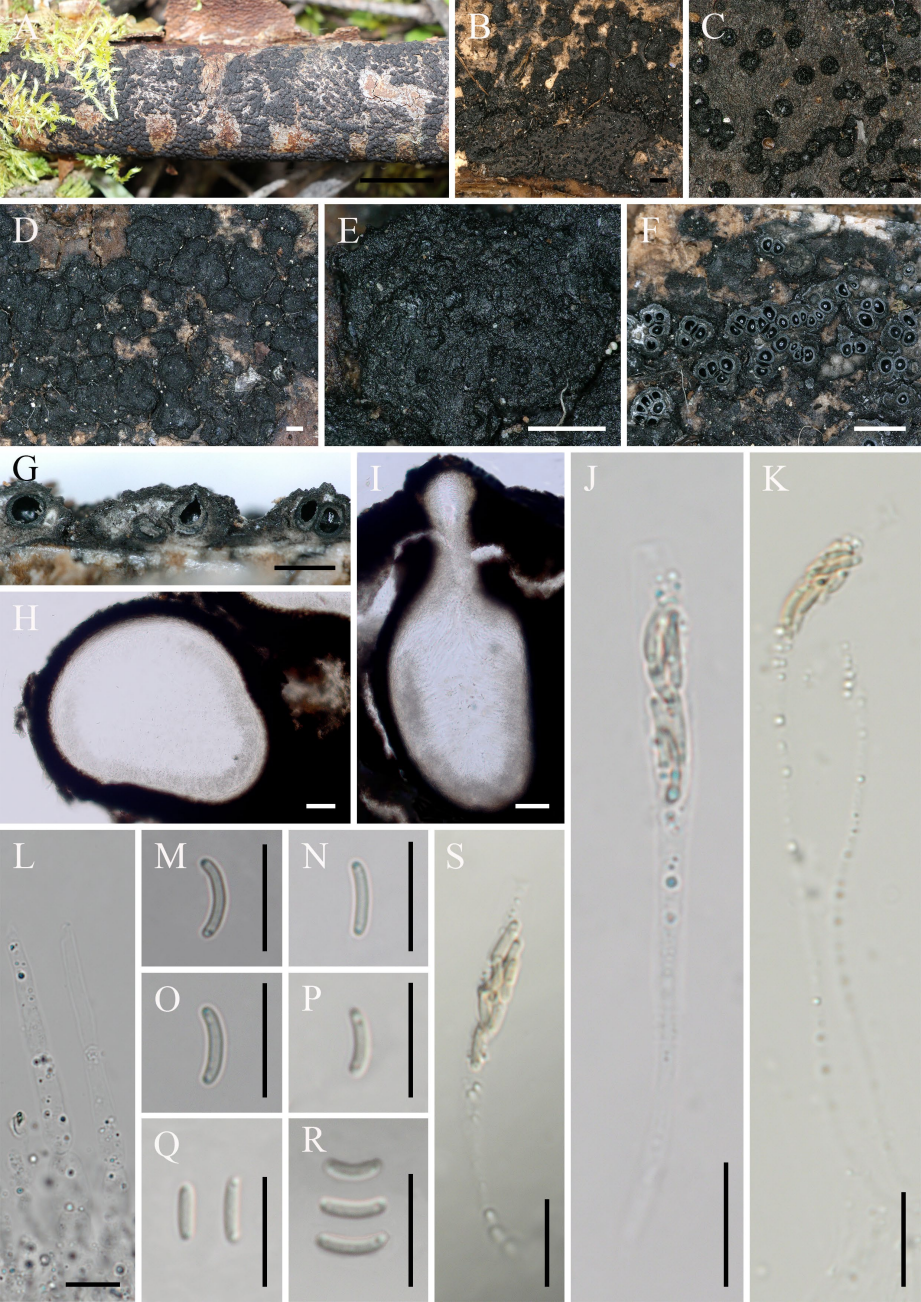
*Diatrype linzhiensis* (FCATAS 4304, Holotype). **(A–E)** Stromata on substrate. **(F)** Cross section of a stroma. **(G,I)** Vertical section through stroma showing ostiole and perithecia. **(H)** Peridium. **(L)** Paraphyses. **(J,K,S)** Asci. **(M–R)** Ascospores. Scale bars: **(A)** = 15 mm; **(B)** = 500 μm; **(C)** = 100 μm; **(D,E,G)** = 500 μm; **(F)** = 1 mm; **(H,I)** = 50 μm; **(J–S)** = 10 μm.

*Diatrype linzhiensis*: Hai X. Ma & Z.E. Yang, sp. *nov.* (Figure 3).

MycoBank: MB 846129.

*Type*: China. Tibet Autonomous Region, Linzhi City, Milin County, Pai Town, 29°30′7′ N, 94°50′33′ E, alt. 1,004 m, saprobic on decaying branches of *Betula* L., 7 October 2021, Haixia Ma, FCATAS 4304 (holotype).

*Etymology*: referring to the locality (Linzhi City) of the type specimens.

*Descriptions*: Saprobic on decaying branches of *Betula* L. Sexual morph: Stromata scattered on the host, irregular in shape, solitary to gregarious, form patchy clumps, cushion-like, superficial, upper surface nearly flat; surface black, with punctiform cone-shaped and sulcate ostioles scattered at surface. Endostroma consists of outer black, small, dense, and an inner layer of white to pale olivaceous gray, large. Perithecium immersed in stroma, globose to subglobose, 222–385 μm high × 164–367 μm diam (x̄ = 294 × 269.6 μm, *n* = 30), with a neck, cylindrical. Peridium composed of outer layer of brown, thin-walled cells, inner layer of hyaline thin-walled cells. Ostiole opening separately, papillate, black. Paraphyses elongate, hyaline, filiform, branched, septate, guttulate. Asci 52–134 × 4.1–7.9 μm (x̄ = 68.2 × 6 μm, *n* = 50), 19–40 × 4.1–7.9 μm in spore bearing part, eight-spored, unitunicate, clavate, long-stalked (27–67 μm), apically flat. Ascospores 5–7.8 × 1–1.4 μm (x̄ = 6.1 × 1.2 μm, *n* = 50), overlapping, allantoid, aseptate, slightly curved, yellowish, rounded ends with two guttules, smooth-walled.

Asexual morph: Undetermined.

*Additional specimen examined*: China. Tibet Autonomous Region, Linzhi City, Milin County, Pai Town, 29°30′7′ N, 94°50′34′ E, alt. 990 m, saprobic on decaying branches of *Betula*, 7 October 2021, Haixia Ma, FCATAS 4381.

Note: *Diatrype linzhiensis* is characterized by cushion-like stromata superficial, solitary to gregarious, form patchy clumps, flat, black, globose to subglobose perithecium with a neck immersed in stroma, hyaline paraphyses long filiform, branched, septate, eight-spored asci with apically flat, yellowish ascospores allantoid to slightly curved. The new species was found on branch of *Betula* sp., *D. albopruinosa* (Schwein.) Cooke, *D. betulae* H.Y. Zhu & X.L. Fan, *D. oregonensis* (Wehm.) Rappaz and *D. stigma* (Hoffm.) Fr. were also reported on *Betula* sp. (Tiffany and Gilman, 1965; Rappaz, 1987; Trouillas et al., 2010b; Vasilyeva and Ma, 2014; Zhu et al., 2021). However, *D. albopruinosa* differs in its larger ascus (40–60 × 10–15 μm) and ascospores (12–15 μm; Vasilyeva and Ma, 2014); *D. betulae* has no sexual morph to be observed (Zhu et al., 2021); *D. oregonensis* differs from *D. linzhiensis by* larger ascus (50–65 × 6–9.5 μm) and ascospores (10–12 × 2–2.5 μm; Trouillas et al., 2010b); *D. stigma* differs in its stromata widely effused and smaller perithecia (150–200 μm; Vasilyeva and Ma, 2014). In the phylogenetic tree (Figure 1), *D. linzhiensis* and *D. undulata* (Pers.) Fr. formed a relatively strongly supported lineage. Morphologically, *D. undulata* differs from *D. linzhiensis* by having dark brown, widely effused stromata, with small stellate ostioles, surrounded by a black line within the substrate, smaller perithecia (150–200 μm vs. 222–384 μm; Vasilyeva and Ma, 2014).

**Figure 4:**
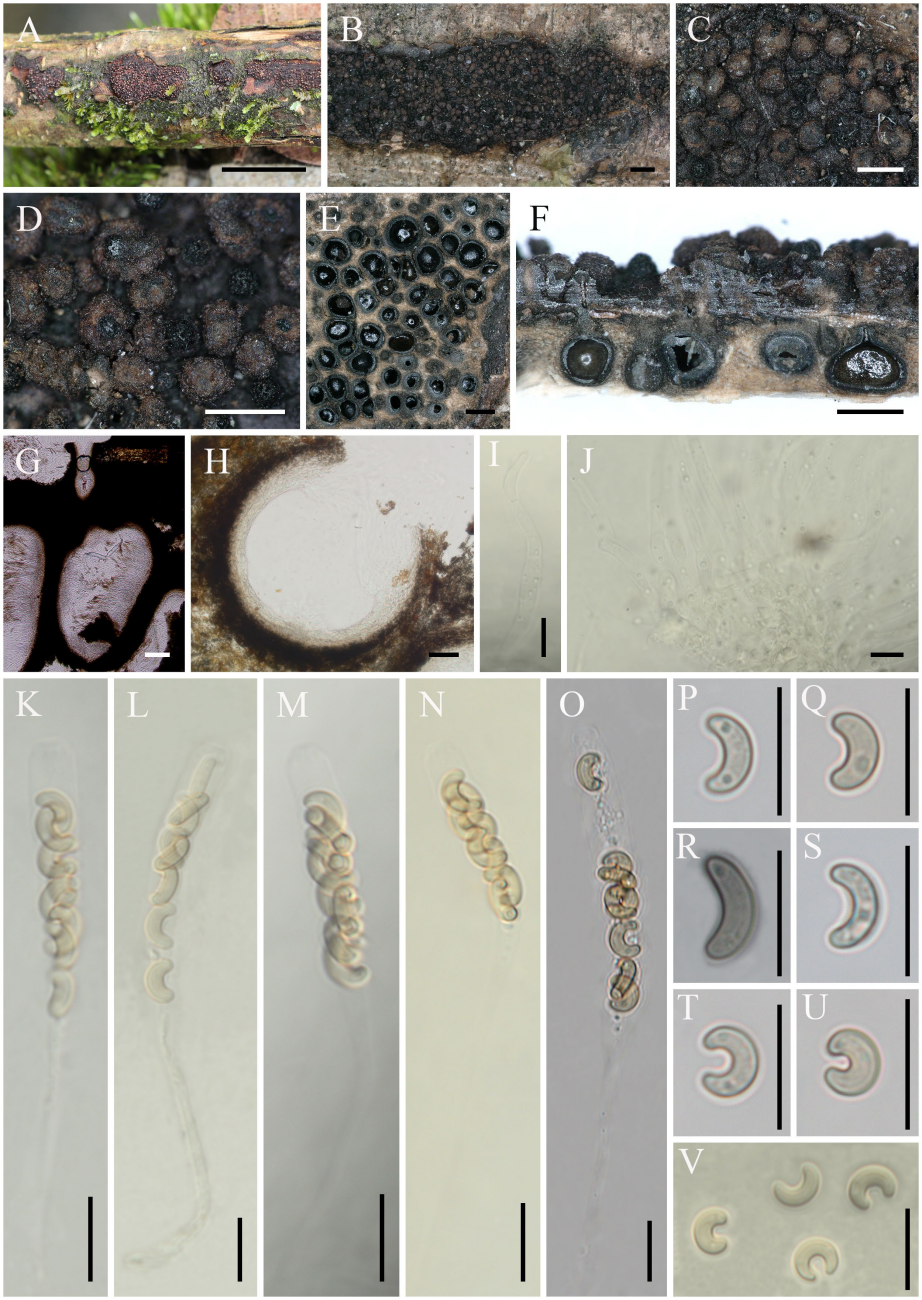
*Eutypella motuoensis* (FCATAS 4082, Holotype). **(A–D)** Stromata on substrate. **(E)** Cross section of a stroma. **(F,G)** Vertical section through stroma showing ostiole and perithecia. **(H)** Peridium. **(I,J)** Paraphyses. **(K–O)** Asci. **(P–V)** Ascospores. Scale bars: **(A)** = 15 mm; **(B)** = 1 mm; **(C–F)** = 500 μm; **(G)** = 100 μm; **(H)** = 50 μm; **(I–V)** = 10 μm.

*Eutypella motuoensis* Hai X. Ma & Z.E. Yang, *sp. nov.* (Figure 4).

MycoBank: MB 846130.

*Type*: China. Tibet Autonomous Region, Motuo County, 29°19′26′N, 95°20′10′E, alt. 996 m, saprobic on the bark of dead branch, 26 September 2021, Haixia Ma, FCATAS 4082 (holotype).

*Etymology*: referring to the holotype locality of species in Motuo county.

*Descriptions*: Saprobic on dead branches of an unidentified plant. Sexual morph: Stromata scattered on the host, erumpent through bark, semi-immersed, 4–38 mm long × 3–9 mm broad, (x̄ = 16.5 × 6.1 mm, *n* = 20), 0.9–1.4 mm thick, irregular in shape, widely effused, upper surface nearly flat; surface saffron to black, cylindrical protrusions of ostioles cover the surface, 227–540 μm high × 281–391 μm diam (x̄ = 331 × 325 μm, *n* = 20). Endostroma consists of outer black, small, dense, and an inner layer of salmon, large. Perithecium immersed in stroma, globose to subglobose, 422–629 μm high × 351–645 μm diam (x̄ = 532.8 × 495.7 μm, *n* = 30), with a neck, cylindrical. Peridium composed of outer layer of brown, thin-walled cells, inner layer of hyaline thin-walled cells. Ostiole opening separately, black. Paraphyses elongate, hyaline, filiform, branched, septate, guttulate. Asci 60–105 × 4.9–6.9 μm (x̄ = 73.1 × 5.9 μm, *n* = 50), eight-spored, unitunicate, subcylindrical, long-stalked (25–74 μm), with rounded apex. Ascospores 6.3–10.6 × 2–2.7 μm (x̄ = 8.4 × 2.3 μm, *n* = 50), overlapping, allantoid to semicircular, sometimes almost forming a circle, aseptate, subhyaline to yellowish, usually with guttules, smooth-walled.

Asexual morph: Undetermined.

*Additional specimen examined*: China. Tibet Autonomous Region, Motuo County, 29°19′26′N, 95°20′10′E, alt. 1,004 m, saprobic on the bark of dead branch, 26 September 2021, Haixia Ma, FCATAS 4379; Motuo County, Yarlung Zangbo River, the large bend of Linduo, 29°19′38′N, 95°20′29′E, alt. 781 m, saprobic on the bark of dead branch, 24 September 2021, Haixia Ma, FCATAS 4035, FCATAS 4378.

Note: *Eutypella motuoensis* differs from most known species of *Eutypella* and related genera by cylindrical protrusions of ostioles cover the surface and subhyaline to yellowish, semicircular to almost circular allantoic ascospores. Morphologically, *Eutypella semicircularis* S. Chacón & M. Piepenbr., *Eutypa crustata* (Fr.) Sacc., *Echinomyces obesa* (Syd. & P. Syd.) Rappaz, and *Diatrype falcata* (Syd. & P. Syd.) Sacc. are similar to *El. motuoensis* by sharing allantoid to semicircular ascospores. However, *El. semicircularis* differs in its mature urn-shaped ascus and smaller reddish-brown ascospores (4.5–7(−11) × 1.5–2(−2.5) μm; Chacón et al. 2013); *Eutypa crustata* differs from *El. motuoensis* by having smaller perithecia (300–450 μm) and smaller ascus (20–35 × 6–8 μm; Rappaz, 1987); *Echinomyces obesa* is separated from *El. motuoensis* by smaller ascus (10–15 × 4–5 μm) and ascospores (3.5–7.5 × 1.2–1.5 μm; Rappaz, 1987); *Diatrype falcata* differs in its less prominent ostioles, smaller perithecia (250–350 μm), smaller ascus (20–25 × 4–5 μm), and ascospores (5.8–7.5 × 1.2–1.5 μm; Rappaz, 1987). In the phylogenetic tree, *El. motuoensis* is sister to *El. persica* Mehrabi, Asgari & Hemmati, though their relationship is not strongly supported. Morphologically, *El. persica* differs from *El. motuoensis* by its allantoid, slightly curved, hyaline, and smaller ascospores (5–7 × 1.5–2.5 μm; Mehrabi et al., 2019).

## Discussion

The species diversity, taxonomy, and phylogeny of diatrypaceous fungi were intensively studied recently by many authors, and a large number of new taxa were described (Mehrabi et al., 2019; Konta et al., 2020; Dayarathne et al., 2020a,b; Dissanayake et al., 2021; Long et al., 2021; Peng et al., 2021; Zhu et al., 2021; Yang et al., 2022). This study furthers the knowledge of these fungi with the addition of a new genus, three new species, and a new combination in the Diatrypaceae. Morpho-molecular analyses confirmed the introduction of the newly described genus, *Alloeutypa*, for accommodating the new species *A. milinensis* and the new combination *A. flavovirens*. Our phylogenetic analyses on the species of *Diatrype* and *Eutypella* also confirmed that they are all polyphyletic genera, agreeing with the previous studies (Acero et al., 2004; Trouillas et al., 2011; Mehrabi et al., 2019; Konta et al., 2020; Dayarathne et al., 2020a,b; Long et al., 2021; Zhu et al., 2021).

In our phylogenetic trees, most taxa of *Diatrype* (*Diatrype sensu stricto*) formed a main clade with high support values (Figure 1), including *D. disciformis*, the type species of the genus. The new species, *D. linzhiensis,* from China also was included in this group. *Diatrype enteroxantha* (Sacc.) Berl. and *D. whitmanensis* J.D. Rogers & Glawe both formed a single clade in phylogenetic trees but the studied sequences of the two species are not their type specimens. While other taxa, for *D. lancangensis* S.H. Long & Q.R. Li, *D. lijiangensis* Thiyagaraja & Wanasinghe, and *D. palmicola* Jian K. Liu & K.D. Hyde formed a single clade or mixed with clades of other genera, and there are no distinct morphological characteristics to divide them into small genera at present.

In the molecular analyses of ITS and β-tubulin sequences performed by Zhu et al. (2021), *Eutypa flavovirens* (Pers.) Tul. & C. Tul. grouped in a clade with two *Cryptosphaeria* taxa by no supported values. In our analyses (Figure 1), *E. flavovirens* appeared in a strongly supported clade along with the new species *A. milinensis*, suggesting the new species is closely related to *E. flavovirens*. The novel diatrypacous genus, *Alloeutypa*, is therefore introduced in the present study and will help to stabilize the classification of Diatrypaceae. However, the other species of *Eutypa* formed two distinct clades in the family and the generic position remains unresolved, which may need to be studied in the future.

The *Eutypella* species analyzed were distributed in two main separate clades (El *sensu stricto* and El 1), one mixed with taxa of *Paraeutypella* and *Allocryptovalsa* (El 1) and another related to a species of *Anthostoma* (*Eutypella sensu stricto*). *Eutypella motuoensis* formed a sister subclade with *El. persica* with no support values.

The molecular evidence has brought significant changes and increased our understanding of the taxonomy and phylogeny of Diatrypaceae. However, the phylogenetic trees show that the classification of these diatrypaceous fungi in many genera is confusing. To determine more important and useful morphological characteristics for distinguishing those species and to resolve infra-genera and infra-specific phylogeny, more specimens of these species from their original regions and more taxa from other regions should be included in future phylogenetic studies.

## Data availability statement

The datasets presented in this study can be found in online repositories. The names of the repository/repositories and accession number(s) can be found at: https://www.ncbi.nlm.nih.gov/genbank/, ITS (OP538689–OP538696) and TUB2 (OP557595–OP557600) https://www.mycobank.org/page/Home/MycoBank, MycoBank (846109, 846111, 846128, 846128–846130).

## Author contributions

Z-KS, A-HZ, ZQ, and H-XM prepared the samples. Z-EY made morphological examinations and performed molecular sequencing. A-HZ performed phylogenetic analyses. Z-EY and H-XM wrote the manuscript. A-HZ revised the language of the text. H-XM conceived and supervised the manuscript. All authors contributed to the article and approved the submitted version.

## Funding

The study was supported by the National Natural Science Foundation of China (No. 31770023 and 31972848) and the Central Public-Interest Scientific Institution Basal Research Fund for Chinese Academy of Tropical Agricultural Sciences (No. 1630032022001, 1630052022003).

## Conflict of interest

The authors declare that the research was conducted in the absence of any commercial or financial relationships that could be construed as a potential conflict of interest.

## Publisher’s note

All claims expressed in this article are solely those of the authors and do not necessarily represent those of their affiliated organizations, or those of the publisher, the editors and the reviewers. Any product that may be evaluated in this article, or claim that may be made by its manufacturer, is not guaranteed or endorsed by the publisher.

## Footnotes

^1^ http://www.ncbi.nlm.nih.gov/genbank
